# Attitudes to and experiences of intimate partner violence among Rohingya women who married before eighteen years of age

**DOI:** 10.1080/16549716.2021.1943852

**Published:** 2021-07-29

**Authors:** M. Mofizul Islam, Md Nuruzzaman Khan, Md Mashiur Rahman

**Affiliations:** a Department of Public Health, La Trobe University, Melbourne, Australia; b Department of Population Sciences, Jatiya Kabi Kazi Nazrul Islam University, Dhaka, Bangladesh; c Independent Researcher, Bangladesh

**Keywords:** Rohingya, child marriage, intimate partner violence, domestic violence, refugee

## Abstract

Currently, around a million Rohingya refugees live in Cox’s Bazar, Bangladesh. This study examines the attitudes toward physical abuse and experiences of intimate partner violence (IPV) of Rohingya refugee women who experienced child marriage. A cross-sectional survey was conducted in the Rohingya refugee settlement at Cox’s Bazar, Bangladesh. Attitudes towards physical abuse have been assessed by a set of five questions that asked the situation under which ‘hitting or beating’ one’s wife is justifiable. Multivariable logistic regressions are used to examine the associations of exposure to child marriage with (i) attitudes towards the justification of physical abuse by one’s husband and (ii) experiences of IPV in the 12 months prior to the survey. Data are available for 486 participants. Overall, 61.32% of women experienced child marriage (married before 18 years of age) and they were more likely to have strongly justified beatings/hitting one’s wife under certain circumstances (Adjusted Odds Ratio (AOR) = 2.71; 95%CI: 1.78, 4.11), and to have experienced such IPV by their husbands in the 12 months prior to the survey (AOR = 1.72; 95%CI: 1.13, 2.61). These AORs are higher for women married at ages 12–14 than those married at 15–17. Having some formal education among husband and wife is protective of abuse within a marriage. Rohingya women’s attitudes towards and experiences of IPV are associated with their exposure to child marriage. Interventions for stopping child marriage, marriage registration, social support group, and legal interventions are needed. Offering formal education to all children needs to be prioritized.

## Background

Child marriage is a pervasive harmful practice and public health concern [1]. Although boys are sometimes subjected to child marriage, girls are disproportionately affected [1]. Research suggests that child marriage places young girls at elevated risk for intimate partner violence (IPV), which could be physical, sexual, and emotional [2]. Although both child marriage and IPV are common in many parts of the world, these practices could be more prevalent in refugee settings [3,4]. However, these practices may go unreported in refugee settings, particularly when refugees do not have a recognized legal status [4,5]. Women who experienced child marriage may be enduring IPV for a long time and may consider it normal or too late to do anything. If they notice an exacerbation, they may not prioritise it to do anything about it, particularly in a displacement crisis. Although there is growing attention to child marriage and IPV globally, research on these practices in refugee settings is scarce.

This short communication examines the attitudes toward and experiences of IPV of Rohingya refugee women who were married before the age of 18. In 2017, Bangladesh allowed around 700,000 Rohingya refugees from Myanmar to cross the border and settle in its southern district of Cox’s Bazar. These people fled an ethnic cleansing, which was declared a genocide by the International Court of Justice [6]. Combining 300,000 refugees who fled previously [7], currently almost one million Rohingya refugees live in congested makeshift shelters. They are identified as ‘Forcibly Displaced Myanmar Nationals’. This designation puts Rohingya people on precarious legal footing under domestic law and denies legal rights that would attach to refugee status [8]. The existing legal protection is ambiguous and does not offer them freedom of movement and employment. They live on food and other support provided by the World Food Program, the UN Refugee Agency and other development partners. The overall circumstances may make Rohingya girls vulnerable to child marriage and put women at an increased risk of IPV.

## Methods

Data of this study come from a cross-sectional survey that we conducted during November 2019 in Kutupalong Refugee makeshift. Around 600,000 Rohingya people live there. This makeshift was built immediately after the arrival of the 2017 influx [8]. The sample is Rohingya women who had become married or given birth after moving to Bangladesh. Six female interviewers who lived in nearby communities conducted the survey either in participants’ houses or outside, without the presence of others. To ensure this, the interviewers had to make several visits to the shelters of some participants. They endeavored to interview as many eligible women as possible during the survey-week. After removing the outliers and missing values, data are available for 486 participants who satisfied the inclusion criteria and attended the survey. The participation rate is 90%.

### Exposure variable

Marriage before the age of 18 is the exposure variable, which is determined by the response to the age at first marriage of the participants.

### Outcome variables

Participants’ attitudes towards wife-beatings, experiences of beating/hitting by their husbands and sexual intercourse with their husbands against their desire in the 12 months prior to the survey are the outcome variables.

We developed composite scores for measuring women’s attitudes towards justification of hitting/beatings one’s wife in following five circumstances: Do you think it is justified for a husband to beat/hit his wife if she 1) goes out without telling her husband; 2) argues with her husband; 3) neglects her children; 4) refuses to have sex with her husband; and 5) burns food while cooking. For each of these questions, response options were yes (score = 1), don’t know (score = 0), and no (score = ̶ 1). These questions were adopted from the Demographic and Health Survey, which is validated and recognized worldwide [9]. Composite scores were computed for each respondent based on the average of responses to the number of questions [10]. For instance, if somebody responded to four questions, the average score was computed for that four times only. Therefore, a high average score indicates high justification, and a low average score indicates little justification for hitting/beatings. Women who scored more than the mean score of the sample were identified as strongly justifying and those who scored less than the mean score were identified as weakly justifying the hitting/beatings one’s wife in these circumstances. Women were also asked whether their husbands beat/hit them or if they had unwanted sexual intercourse with their husbands in the last 12 months.

### Analysis

We use descriptive statistics to characterize the demographic profile and to estimate the prevalence of exposure, and outcome variables. Multivariable logistic regressions are used to examine the associations between (i) exposure to child marriage and attitudes towards the justification of physical abuse by one’s husband under certain circumstances, and (ii) exposure to child marriage and experiences of IPV.

## Results

**Figure 1.**
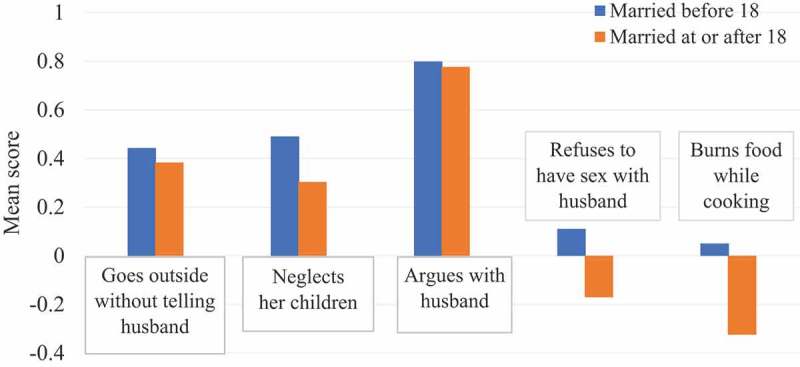
Women’s perceptions of the situations justified for beating or hitting by one’s husband

The average age of 486 participants at the time of the survey was 23.17 years (SD ±5.01) for women and 28.50 years (SD ±7.77) for their husbands. Two-thirds of women attended Madrasahs that teach the Quran, Islamic way of life, and in some cases provide basic primary education and 30% received some formal education. The educational profile of their husbands is similar. Overall, 61.32% (298/486) of women experienced child marriage, and 22.82% (68/298) of them was married after being displaced to Bangladesh. The youngest age at child marriage was 12 years. The mean composite scores for all five questions are higher for these women (Figure 1) than those who were married at or after 18, meaning that the former group is more accepting than the latter towards the justification of beatings/hitting a wife by his husband in certain circumstances. The difference in mean scores between these two groups is minimum for arguing with husband and maximum for burning food while cooking. More women with a history of child marriage experienced beatings/hitting by their husbands (76.51%, 228/298) than those without such a history (65.43%, 123/188). Similarly, more women with a history of child marriage experienced unwanted sex with their husbands (59.40%, 177/298) than those without such a history (54.26%, 102/188).

Women who were married before the age of 18 were significantly more likely to have strongly justified beatings/hitting one’s wife under these five circumstances (AOR = 2.71; 95%CI: 1.78, 4.11) and experienced such IPV by their husbands during 12 months prior to the survey (AOR = 1.72; 95%CI: 1.13, 2.61) (Table 1). The AOR for justification of beatings/hitting is 4.33 (95%CI: 2.42, 7.74) among women married at ages 12–14 and 2.19 (95%CI: 1.39, 3.44) among those married at ages 15–17 (results not shown in the table). The AORs for experiences of beatings/hitting are 1.95 (95%CI: 1.06, 3.58) and 1.62 (95%CI: 1.02, 2.57) for these two subgroups of women, respectively. There is no significant association between the history of child marriage and the experiences of unwanted sexual intercourse with the husband. The higher the age, the more lenient they are towards the five circumstances of physical IPV by their husbands. Having some formal education is protective of experiencing beatings/hitting and unwanted sexual intercourse by their husbands during the 12 months before the survey (Table 1). Also, women whose husbands received some formal education are less likely to hold a strong justification towards beatings/hitting one’s wife under five circumstances (AOR = 0.39; 95%CI: 0.26, 0.58).

**Table 1.** Multivariable logistic regression examining attitudes to and experiences of intimate partner violence of Rohingya refugee women who were exposed to child marriage

Independent variable	Strongly justified beatings/hitting		Experienced beating/hitting by husband in twelve months prior to the survey		Had to have unwanted sex with husband in the twelve months prior to the survey	
	AOR (95% CI)	*p*	AOR (95% CI)	*p*	AOR (95% CI)	*p*
Married before the age of 18						
No (reference)	**1**		**1**		1	
Yes	**2.71 (1.78, 4.11)**	**<0.01**	**1.72 (1.13, 2.61)**	**0.01**	1.29 (0.87, 1.91)	0.19
Women’s age	**1.11 (1.04, 1.19)**	**<0.01**	1.02 (0.95, 1.09)	0.65	1.01 (0.95, 1.08)	0.76
Husbands’ age	0.98 (0.94, 1.03)	0.47	1.00 (0.96, 1.04)	0.93	1.02 (0.97, 1.06)	0.44
Woman received at least some formal education						
No (reference)	1		**1**		**1**	
Yes	0.96 (0.59, 1.55)	0.86	**0.54 (0.31, 0.94)**	**0.03**	**0.59 (0.36, 0.95)**	**0.03**
Husband received at least some formal education						
No (reference)	**1**		1		1	
Yes	**0.39 (0.26, 0.58)**	**<0.01**	1.31 (0.86, 2.00)	0.21	1.26 (0.86, 1.86)	0.24

AOR: Adjusted Odds Ratio; CI: Confidence Interval; Numbers marked in bold indicate statistically significant results.

## Discussion

The findings suggest child marriage and IPV are highly prevalent in the Rohingya community, and women who married before 18 were more likely than others to have experienced physical abuse by husbands and to believe that a man is justified in beating his wife in certain circumstances. While much of our findings are consistent with the literature [4,11], what is concerning is that the baseline prevalence of child marriage and IPV had been already entrenched in this community before displaced to Bangladesh [4]. The situation may be aggravated due to perceived insecurity in a foreign country, ambiguous legal footing, lack of access to formal education, social norms, religious beliefs, patriarchy, and poverty [12,13]. However, they may not do something about it, even if they only start experiencing IPV or notice that it has exacerbated after displacement because they are in an emergency situation.

Literature suggests that among women of age 20–24 years, physical, sexual, and emotional abuse by intimate partners are strongly connected to child marriage [14]. One possible explanation for justifying physical abuse and experiencing such abuse by women with a history of child marriage is ‘perceived normalcy’ to this practice. Perhaps, most of them have been enduring this for a long time. The difference in socio-demographic backgrounds between those who were married as children and those as adults may be a major factor. A previous study found that women married before the age of 18 had lower levels of education, were more likely to endorse patriarchal gender norms and had lower levels of autonomy within the household [15]. Also, host country Bangladesh has one of the world’s highest rates of child marriages among women of 20–24 years old [16]. Further research is needed to examine the influence of this high rate on child marriage practice in the Rohingya community.

Our results highlight the importance of having a formal education, as it was found protective for IPV, and women’s less accepting attitudes towards physical abuse, if husbands received some formal education. Although currently, an opportunity for education is limited, the good news is that in early 2020, the government of Bangladesh granted permission to young Rohingya refugees to have formal schooling [17].

This study found that the higher the age of women the greater the tendency to justify hitting/beatings by one’s husband under certain circumstances. This observation is inconsistent with some previous findings [18] but consistent with others [19]. A possible explanation for this is more conforming attitudes of women of relatively high age towards patriarchal norms and values. Also, younger women are more likely to have some formal education. On the other hand, relatively high odds ratios for strong justifications towards and experiences of beatings/hitting among those married at ages 12–14 may be due to less educational attainment and hence less power, status, agency and autonomy [20]. Marriage often marks the end of a girl’s education [20].

Despite some limitations such as generalizability, social desirability and response biases, this study-findings have important implications. We acknowledge that basic subsistence for such a large population is a priority and a massive undertaking for the Government of Bangladesh, national and international development partners. However, child marriage and IPV are not something that can be put aside. Also, addressing these issues may reduce unintended pregnancy [21] and the demand for health care and social services. Intervention for stopping child marriage, compulsory birth and marriage registration, all-women support group, and legal support are needed [22]. Offering formal education to all children needs to be prioritized. Finally, we recommend future research to have an in-depth qualitative understanding of underlying factors of child marriages in the Rohingya community.

## Paper context

Child marriage is common in the Rohingya community. But the attitudes towards and experiences of intimate partner violence among Rohingya women who married before 18 years of age remained unknown. Our results demonstrate that these women are more likely than others to believe that men are justified in beating their wives in certain circumstances, and more likely to have experienced such violence. Compulsory marriage registration, legal support, and formal education for children are policy recommendations.
